# E Protein Transcription Factors as Suppressors of T Lymphocyte Acute Lymphoblastic Leukemia

**DOI:** 10.3389/fimmu.2022.885144

**Published:** 2022-04-20

**Authors:** Geoffrey Parriott, Barbara L. Kee

**Affiliations:** ^1^ Committee on Immunology, University of Chicago, Chicago, IL, United States; ^2^ Committee on Cancer Biology, University of Chicago, Chicago, IL, United States; ^3^ Department of Pathology, University of Chicago, Chicago, IL, United States

**Keywords:** Leukemia, E protein, TAL1, LYL1, murine, T lymphocyte

## Abstract

T Lymphocyte Acute Lymphoblastic Leukemia (ALL) is an aggressive disease arising from transformation of T lymphocytes during their development. The mutation spectrum of T-ALL has revealed critical regulators of the growth and differentiation of normal and leukemic T lymphocytes. Approximately, 60% of T-ALLs show aberrant expression of the hematopoietic stem cell-associated helix-loop-helix transcription factors TAL1 and LYL1. TAL1 and LYL1 function in multiprotein complexes that regulate gene expression in T-ALL but they also antagonize the function of the E protein homodimers that are critical regulators of T cell development. Mice lacking E2A, or ectopically expressing TAL1, LYL1, or other inhibitors of E protein function in T cell progenitors, also succumb to an aggressive T-ALL-like disease highlighting that E proteins promote T cell development and suppress leukemogenesis. In this review, we discuss the role of E2A in T cell development and how alterations in E protein function underlie leukemogenesis. We focus on the role of TAL1 and LYL1 and the genes that are dysregulated in *E2a^-/-^* T cell progenitors that contribute to human T-ALL. These studies reveal novel mechanisms of transformation and provide insights into potential therapeutic targets for intervention in this disease.

## Introduction

T cell acute lymphoblastic leukemia (T-ALL) is an aggressive disease that accounts for 15% of pediatric and 25% of adult leukemia cases (1). These leukemias can be grouped into subtypes based on their unique gene expression profiles and response to therapy with subtypes mirroring known stages of T cell development (2–4). Consistent with these heterogeneous phenotypes, mutations in, or altered expression of, genes encoding multiple transcription factors (*cMYC*, *IKZF1*, *GATA3*, *TCF7*, *LEF1*), epigenetic regulators (*EZH2* and *PHF6*), cytokine receptors (*IL7R* and *FLT3*), cell cycle regulators (*CDKN2A* and *CDKN2B*), and signaling proteins (PTEN) have been identified in subsets of human T-ALL (5–7). Despite this heterogeneity, there are oncogenic pathways that are dysregulated broadly across multiple T-ALL subtypes. In particular, mutations that impact the Notch signaling pathway are present in approximately 80% of T-ALL (8). Approximately 60% of T-ALL cases also have mutations that augment expression of the transcription factors T acute lymphoblastic leukemia antigen 1 (TAL1), also called Stem Cell Leukemia (SCL), and lymphocytic leukemia antigen 1 (LYL1) (9). The high frequency of NOTCH1, TAL1 and LYL1 dysregulation indicate that these pathways impact key processes underlying T cell homeostasis at multiple stage of development and make them attractive targets for therapeutic intervention. Indeed, NOTCH signaling inhibitors have been designed and tested in clinical trials and show some utility for treatment of T-ALL (10). Unfortunately, the broad expression of NOTCH1 has limited the utility of NOTCH1 inhibition as a single agent for treatment of T-ALL, although recently described inhibitors have had some success (10). Nonetheless, ongoing studies taking advantage of partial Notch signaling inhibition combined with targeting other essential pathways is a promising approach for future therapeutics (11). A better understanding of the mechanisms leading to leukemogenesis and the sensitivity of leukemias to inhibition of oncogenic pathways will help to develop novel therapeutics to intervene in this disease.

TAL1 and LYL1 are basic helix-loop-helix (bHLH) proteins that bind DNA in association with the E protein transcription factors (12). The E proteins form homodimers that are critical for B and T lymphocyte development and dimerization with TAL1 or LYL1 alters E protein DNA binding specificity and promotes interactions with unique transcriptional complexes (13–15). Therefore, while TAL1:E protein or LYL1:E protein dimers in T-ALL regulate leukemia-associated genes, expression of TAL1 and LYL1 can also interrupt the function of E protein homodimers. There is substantial evidence in mice to suggest that inhibition of E protein function is sufficient to promote T cell progenitor transformation (16–19). These mouse models recapitulate features of their human T-ALL counterparts such as recurrent mutations in the *Notch1* gene and a requirement for Notch signaling for their survival (20–22). In this review we will discuss the mechanisms driving the genesis and maintenance of T-ALL with a focus on the insights gained through studies in *E2a*-deficieint mice.

## E2a Proteins in Lymphopoiesis

The *Tcf3* (*E2a)* gene encodes 2 bHLH proteins (E12 and E47) through alternative splicing of exons encoding the bHLH domain (13). The HLH domain is involved in dimerization with other HLH proteins and the basic region is largely responsible for DNA binding, although some DNA contacts are made with the HLH domain (12). The bHLH domains of E12 and E47 share approximately 80% identity and they bind the same DNA motif, although with differing affinity, and interact with the same proteins (23, 24). There are two additional genes encoding for E proteins in humans and mice, *TCF12* (HEB) and *TCF4* (E2-2), that each code for two E box binding proteins through alternative transcription start sites, resulting in proteins with differing activation domains but identical bHLH domains (25). Other proteins that dimerize with E proteins include the Class IV HLH proteins (ID1-4), which lack a DNA binding domain and therefore prevent E proteins from stably binding DNA, and Class II bHLH proteins, which are largely cell type specific (i.e. MYOD in muscle cells and TAL1 in hematopoietic stem cells) (12). E proteins are broadly and constitutively expressed and are generally found in complexes with tissue-restricted Class II proteins. However, E proteins function as homodimers in lymphocytes; E2A homodimers predominating in B lymphocytes and dimers of E2A and HEB being prevalent in T lymphocytes (13, 25). Consistent with this, *E2a^-/-^* mice have severe defects in lymphopoiesis with a complete lack of B lymphocytes and a 3-5X decrease in thymocyte numbers prior to the onset of leukemogenesis (16, 17, 26, 27). *Tcf12-*deficiency or *Tcf4-*deficiency also impacts T cell development but to date, neither of these deficiencies is sufficient to promote T-ALL like disease (25, 28–30).

**Figure 1:**
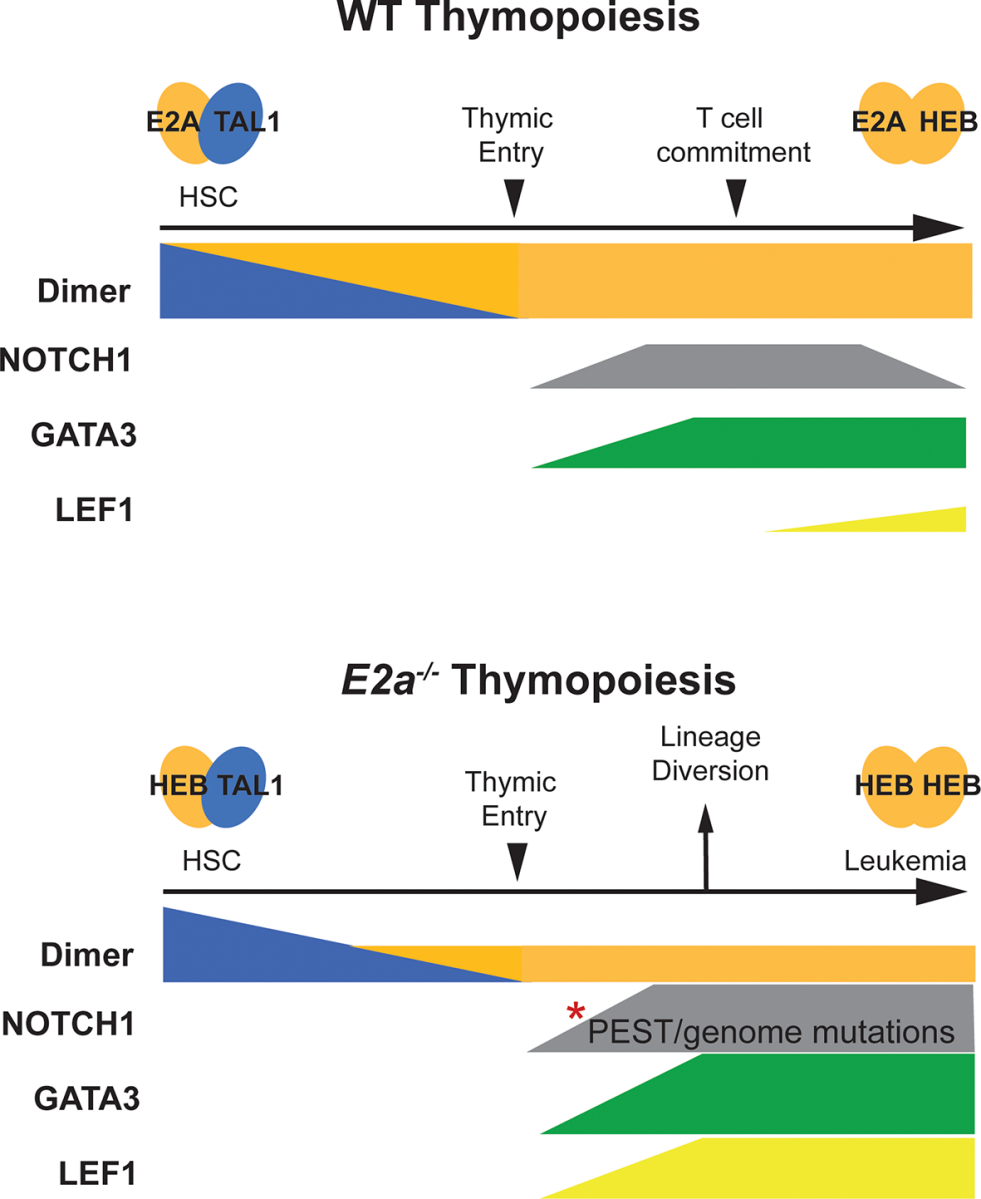
Schematic representation of T cell development in WT and *E2a^-/-^* mice. T cell development in WT mice is depicted in the top half of the figure. Tal1:E2A (or E protein) dimers are prevalent in HSCs and decline as progenitors differentiate toward the T cell lineage. At the time of thymic commitment E2a:HEB dimers dominate. NOTCH1 and GATA3 start to be expressed upon thymic entry, along with TCF1 (not depicted) under the influence of NOTCH1 ligands in the thymus. LEF1 expression initiates with commitment to the T lymphocyte lineage but remains low throughout T cell development. T cell development in *E2a^-/-^* mice is depicted on the lower half of the figure. Here, Tal1:HEB or E2-2 dimers control early hematopoiesis resulting in a reduced number of thymic seeding progenitors that can only express HEB : HEB or E2-2 dimers after extinction of TAL1 expression. GATA3 expression is high and diverts progenitors toward the ILC/NK fate. Mutations in the *Notch1* gene accumulate and LEF1 is expressed at very high levels. Combined, these alterations in NOTCH1, GATA3 and LEF1 contribute to T cell transformation.

An advantage of studying T-ALL in mice is that we can track progenitors prior to the onset of disease and discern the impact of these mutations on T cell development as well as on leukemogenesis (**Figure 1**). TAL1, and by analogy its E protein partners, plays a critical role in specification of hematopoietic stem cells (HSCs) from hemangiogenic endothelium but TAL1 is not essential for HSC survival after HSC development (31, 32). However, post HSC specification, TAL1 plays important roles in megakaryocyte differentiation and erythropoiesis (33, 34). LYL1 is not essential for HSC specification, although it becomes essential when TAL1 is limiting, indicating that these proteins have some redundant functions (35). ChIP-seq experiments with TAL1 and LYL1 in HSC-like cell lines revealed extensive overlap in their binding sites indicating that these proteins regulate an overlapping set of genes (36). In *E2a^-/-^* mice, HSC specification is intact but lymphopoiesis is impacted at the stage when HSCs become specified to the lymphoid fate with fewer lympho-myeloid primed progenitors (LMPPs) and a failure to initiate expression of multiple lymphoid genes (37, 38). In LMPPs E2A likely functions in cooperation with LYL1 since *Lyl1^-/-^* mice have a strikingly similar phenotype to *E2a^-/-^* mice at this stage (39). In contrast, TAL1 antagonizes T lymphocyte specification within the HSC and LMPP populations (40). Therefore, despite the similarity in TAL1 and LYL1 structure and their overlapping function in HSC specification, these proteins function in an opposing manner to regulate lymphoid specification. Given the roles of E2A, TAL1 and LYL1 in T-ALL, we anticipate that understanding how these proteins control lymphocyte development will provide insights into the mechanisms that drive lymphopoietic alterations and transformation.

E2A proteins are required for proper expression of *Notch1* at the inception of T cell development (37, 41). Consistent with this, when *E2A^-/-^* multipotent progenitors are cultured under T cell differentiation conditions *in vitro* they fail to generate T cells unless they are transduced with a NOTCH1 producing retroviral vector (42). *E2a^-/-^* DN2 thymocytes struggle to enter the T cell lineage and fail to control the expression of GATA3, which is substantially elevated in *E2a^-/-^* DN2 and DN3 thymocytes (43). This elevated expression of GATA3 contributes to diversion of these cells toward the innate lymphoid lineages, which is particularly evident when *E2a* and *Heb* are both deleted or when ID1 is over expressed in T cell progenitors (43–46). Indeed, heterozygous deletion of *Gata3* restores differentiation of *E2a^-/-^* DN2 cells into the T cell lineage (43). Ectopic expression of GATA3 under the control of the CD2 promoter is able to promote T cell transformation suggesting that this failure to repress *Gata3* could be a key event in the generation of *E2a^-/-^* leukemias (47). In established *E2a^-/-^* leukemia lines re-expression of E2A proteins alters the transcription of numerous genes including *Gata3*, which is indirectly regulated by E2A-mediated induction of GFI1B (48). Whether GFI1B, alone or in combination with the related transcription factor GFI1, functions to dampen *Gata3* expression at the inception of T cell development remains to be fully explored but it is notable that both *Gfi1b^-/-^* and *Gfi1^-/-^* mice have defects in T cell development that overlap with those of *E2a^-/-^* mice (49, 50). During B cell development *Gata3* is repressed by EBF1 suggesting that GFI1/GFI1B and EBF1 might play similar roles in progenitors prior to their entry into the T and B cell developmental pathway with EBF1 leading to more severe, or sustained, repression of *Gata3* (51).

The few T cell progenitors that develop from *E2a^-/-^* DN2 thymocytes highly express LEF1, an effector of the Wnt signaling pathway, and LEF1 is required for the survival of *E2a^-/-^* leukemias (52, 53). LEF1 is not essential for T cell development owing to the high expression of the related transcription factor TCF1 in T cell progenitors (54). *Tcf7* (encoding TCF1) is regulated by NOTCH1 and plays a major role in T cell lineage specification (55, 56). TCF1 is expressed in *E2a^-/-^* thymocytes despite the increased expression of LEF1; nonetheless, LEF1 impacts *E2A^-/-^* T cell development. Indeed, deletion of *Lef1* from *E2a^-/-^* T cell progenitors results in a profound loss of DN3 thymocytes while, surprisingly, not affecting overall T cell numbers (53). These findings suggest that LEF1 plays a role in controlling the maturation of *E2a^-/-^* T cells. *Lef1* mRNA is elevated in multiple mouse models that develop T-ALL, and as described later in this review, LEF1 can play both oncogenic and tumor suppressor roles in these models depending on the timing of its expression (53, 57–60). In the following sections we will discuss the known contributions of the E2A interacting proteins TAL1 and LYL1 and the genes dysregulated in *E2a^-/-^* thymocytes to human and murine T-ALL.

## TAL1 and LYL1

**Figure 2:**
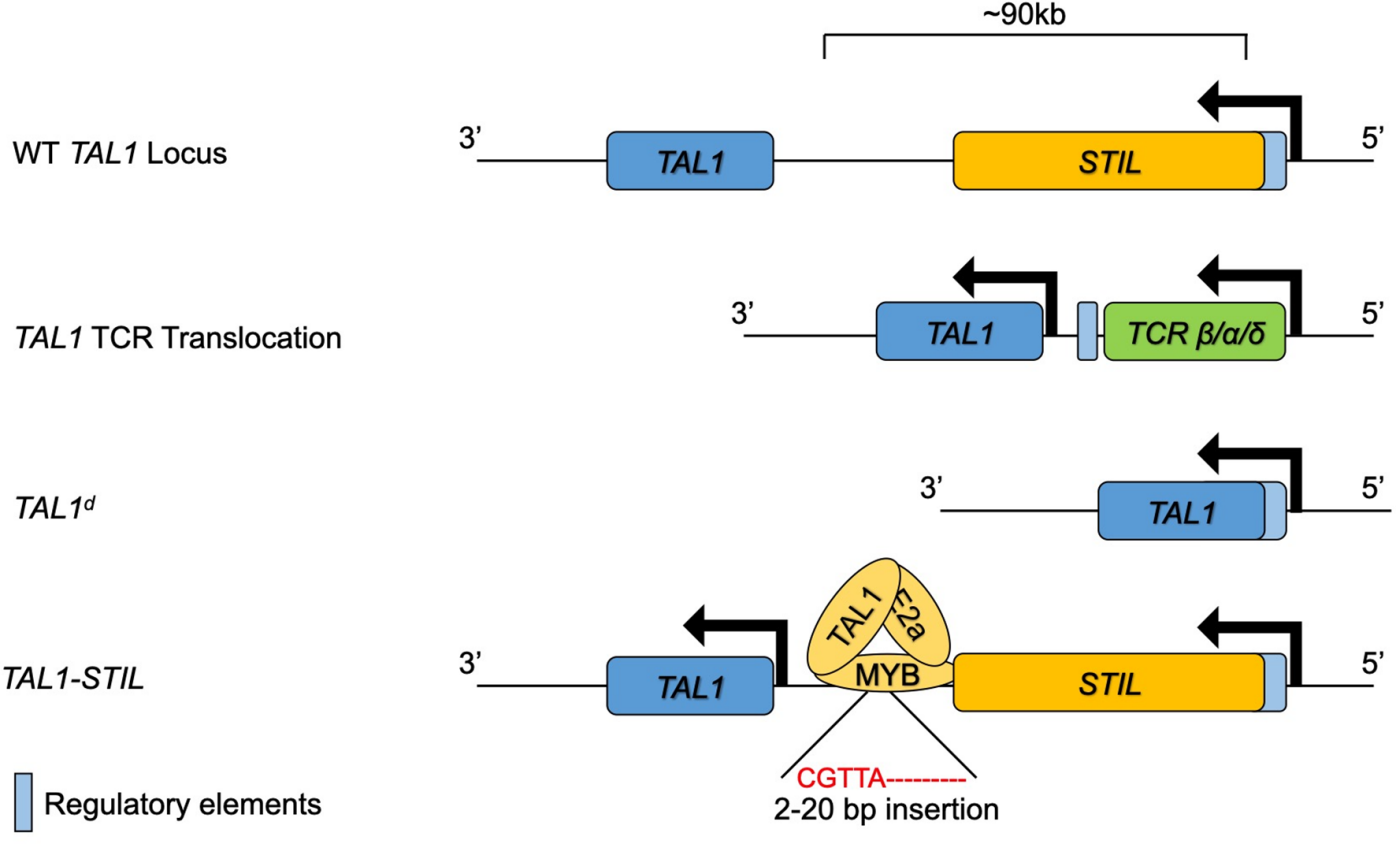
The *TAL1* genomic locus and mutations leading to ectopic expression in T lymphocytes and T-ALL. The WT *TAL1* locus is depicted downstream of the *STIL* gene. *TAL1* transcription is increased in T lymphocytes through genomic translocation into the *TCRB/A/D* locus, through genomic deletions bringing the *STIL* enhancer close to *TAL1* or through insertions that create a novel *MYB* binding enhancer upstream of *TAL1*.

TAL1 was identified as a gene involved in the t(1:14) and t(1;7) chromosomal translocations in T-ALL, which place *TAL1* under the control of the *TCRA*/*TCRD* or *TCRB* locus, respectively (61–63). LYL1 was also identified through a chromosomal translocation in T-ALL in which *LYL1* on chromosome 19 is juxtaposed to the *TCRB* constant regions on Chromosome 7 (64). These translocations are found in approximately 3-7% of TAL1/LYL1^+^ T-ALL cases, however, there are frequent alterations at the *TAL1* locus in T-ALL including deletions such as *TAL1^d^*, which arises from a site-specific DNA recombination event causing a 90kb deletion upstream of *TAL1* (62). These deletions place the coding region of the *TAL1* gene downstream of regulatory elements in the SCL interrupting locus (*STIL*). The *STIL* regulatory elements are constitutively active in thymocytes, resulting in ectopic TAL1 expression. These *TAL1* upstream deletions are specific for T-ALL cells and are most likely caused by erroneous V(D)J recombinase activity (65, 66). Alterations in the *TAL1* gene that result in ectopic T lymphocyte expression of TAL1 are now recognized to be present in as many as 60% of T-ALL (1). While these genomic alterations account for a majority of T-ALL associated *TAL1* expression, a subset of patients have ectopic TAL1 expression without these alterations. Studies into the mechanisms of *TAL1* deregulation in these patients revealed small insertions (<20bp) in a region 8kb upstream of *TAL1* that create a *de novo* MYB binding site that results in strong enhancer activity in these leukemias (67). Chromatin immunoprecipitation followed by high throughput sequencing (ChIP-seq) experiments showed that MYB binds to this novel site along with chromatin remodelers and other components of the DNA transcriptional machinery. Deletion of the novel MYB binding site abrogated MYB binding and significantly reduced TAL1 expression. MYB is highly expressed in thymocytes and MYB is often dysregulated in cancer and thus this mutation can lead to robust *TAL1* transcription in leukemic cells (68). Taken together, these findings outline multiple mechanisms leading to the errant expression of TAL1 in T-ALL (**Figure 2**).

TAL1 positive leukemias frequently express CD4 and CD8 and have a cortical phenotype similar to what is observed in *E2a^-/-^* mice (2, 16, 17, 69). In mice, expression of TAL1 under the control of the Lck promoter, which drives gene expression early in T cell development, is sufficient to predispose mice in T-ALL-like disease (70). That TAL1 functions through sequestration of E protein is implied by studies that showed that TAL1-driven leukemia is not dependent on the DNA binding ability of TAL1 (18). Moreover, transgenic expression of ID1 or ID2, which prevent E proteins from binding to DNA, also predisposes mice to develop a T-ALL- like disease (19, 71). It is also notable that Lck-TAL1 promotes leukemia in a dose-dependent manner that is augmented by deletion of the E proteins encoded by *Heb/Tcf12* indicating that E protein dose is a major determinant of leukemogenesis in this model (18). Analogous to what is observed in Lck-TAL1 transgenic mice, ectopic expression of LYL1 in T cell progenitors blocks the formation of E-protein homodimers, suppresses the expression of E2A-dependent genes, and leads to T-ALL like disease (69, 72). These findings suggest that at least a part of the mechanism through which TAL1 and LYL1 promote leukemogenesis is through inhibition of E protein homodimer function. However, LYL1^+^ and TAL1^+^ leukemias have unique gene expression profiles and LYL1^+^ leukemias tend to be related to immature CD4-CD8- T cell progenitors (2). These observations suggest that LYL1 and TAL1 have unique functions or that they are expressed in different cellular contexts, either distinct stages of development or stages of transformation. Interestingly, nearly 30% of pediatric TAL1^+^ T-ALL patients have heterozygous loss-of-function mutations in *USP7*, a deubiquitinating enzyme that interacts with E proteins, and other leukemia-associated proteins, and is associated with decreased E protein target gene expression and increased cell growth (73). Therefore, there appears to be multiple mechanisms contributing to reduced E protein function in T-ALL.

While inhibition of E protein function is sufficient to predispose T cell progenitors to transformation, TAL1 and LYL1 may contribute to transformation through their participation in transcriptional complexes that activate or inhibit gene expression. In hematopoietic progenitors and T-ALL, both TAL1 and LYL1 bind DNA in large complexes that include the LMO (LIM only), LIM domain binding (LDB1), and GATA protein families (74–78). Indeed, LMO proteins are critical members of the TAL1 complex, acting as bridging factors that connect TAL1 to other DNA binding proteins like GATA1/2/3 (79). This is of particular interest because LMO proteins are overexpressed in approximately 10% of T-ALLs and these leukemias frequently have *TAL1* overexpression (1). In a subset of pediatric (3.7%) and adult (5.5%) T-ALL the *LMO2* gene contains intronic indels that result in *de novo* binding sites for the leukemia-associated transcription factors MYB, ETS1 or RUNX1 and thus dysregulated LMO2 expression (80). Interestingly, both TAL1 and LMO1 or LMO2 are required to induce reporter activity in T-ALL cell lines (81). Experiments in human T-ALL cell lines have been vital to elucidating the core components of the TAL1 complex in leukemia. ChIP-seq in these lines has identified E2A, GATA3, LMO1/LMO2, RUNX1, and MYB as co-bound to TAL1 bound regions suggesting multi-transcription factor complex formation (82). Using siRNA to knock down TAL1 or other members of the TAL1 complex, Sanda et al. identified genes regulated by this complex in leukemias (83). Interestingly, expression of the genes that make up the TAL1 complex is decrease upon TAL1 siRNA knockdown. This finding suggests a positive feed-forward mechanism where the oncogenic TAL1 complex promotes expression of itself, in addition to promoting expression of other known oncogenes. TAL1-dependent genes include *MYB*, which positively regulates cell cycle and anti-apoptotic genes, *TRIB2*, which supports the survival of T-ALL cell lines, and *ARID5*, a gene associated with a variety of leukemias (83–85). Of note, the oncogenic microRNA miR-223 is also decreased after reducing TAL1 and ChIP-seq revealed that the TAL1 complex binds to a putative enhancer near this gene (86).

There is evidence indicating that LYL1 forms oncogenic complexes similar to TAL1. Indeed, Jurkat cells forced to express LYL1, LMO2 and LDB1 induced robust target gene expression that was dependent on LMO2 and LDB1 (77). Further, LMO2 is frequently overexpressed in TAL1 expressing leukemias but in LMO2 transgenic mice TAL1 is dispensable for leukemogenesis (87). In contrast, deletion of *Lyl1* significantly increases leukemia latency in LMO2 transgenic mice suggesting that LYL1 supports transformation. Microarray analysis revealed that LMO2 expressing thymocytes have higher *Lyl1* expression compared to wild type thymocytes indicating a potential feed-forward mechanism reminiscent of the mechanism seen in TAL1 expressing leukemias. Consistent with this idea, the *LYL1* promoter contains ETS and GATA binding sites, which promote the expression of *LYL1* in HSCs and both ETS1 and GATA3 are implicated in T-ALL (88–90). Taken together, these data suggest that LYL1 may function in a manner analogous to TAL1 during T cell progenitor transformation.

## NOTCH1

NOTCH1 is constitutively activated in a majority of T-ALL, including those that overexpress *TAL1*, and in leukemias from *E2a^-/-^* mice (20, 91). NOTCH1 functions as both a surface receptor and transcription factor that is essential for T cell development (8, 92). The ligands for NOTCH1 are members of the Jagged and Delta-like family, with DELTA-LIKE 4 (DLL4) being the most important in the thymus (93). NOTCH1 is translated as a single protein that is cleaved in the Golgi to create extracellular and intracellular components that are held together in the membrane by heterodimerization domains (HD) (8). Upon ligand binding the extracellular portion undergoes a conformational change that allows cleavage by a disintegrin and metalloprotease, which exposes a cleavage site for γ-secretase, which then cleaves and liberates the intracellular domain of NOTCH1 (called ICN). The ICN translocates to the nucleus, where it complexes with the DNA bound transcriptional repressor CBF1/RBP-Jκ and recruits Mastermind (MAML) proteins to initiate the transcription of multiple genes that promote T cell specification (8). Activation of NOTCH1 is transient owing to the presence of a PEST sequence at the 3’ end that is recognized by the FBW7 ubiquitin ligase and targets ICN for proteasomal degradation (94). Targets of the ICN/CBF1/MAML complex in murine T cells include *Hes1* and *Tcf7*, both of all of which play critical roles in T cell development (56, 95, 96). Interestingly, Notch signaling also shuts down transcription of *Ccr9*, which encodes a chemokine receptor involved in thymic homing of LMPPs (97, 98). Repression of *Ccr9* may trap lymphoid progenitors in the cortex of the thymus as they undergo commitment to the T cell lineage. Repression of *Ccr9* may also explain why ectopic expression of ICN in murine HSCs promotes T cell transformation without accumulation of these leukemic cells in the thymus (99).

NOTCH1 was identified as an oncogene in T-ALL by its involvement in a t(7;9)(q34;q34.3) translocation that placed the 3’ end of the *NOTCH1* gene under control of the *TCRB* locus, resulting in constitutive activation of NOTCH1 in T cell progenitors (100). This translocation is present in approximately 2% of leukemias, however, it is now appreciated that > 60% of all human T-ALLs have mutations in *NOTCH1* (8). These mutations cluster in the heterodimerization domain (HD) and in the PEST domain. Mutations affecting the HD domains prevent the association of the extracellular and intracellular portions of NOTCH1 thus allowing for spontaneous γ-secretase mediated cleavage to produce active ICN. The PEST domain mutations promote stabilization of ICN by removing the phosphorylation sites that lead to docking of FBW7. These mutations are not mutually exclusive, with ~20% of human leukemias having mutations in both domains (101). Additional mutations have been identified that inactivate FBW7 resulting in the constitutive stabilization of ICN (8, 102, 103). The TAL1 complex also represses *FBWX7* through miRNA-223 suggesting that there may be numerous mechanisms contributing to stabilization of NOTCH1 in T-ALL (86).

Leukemias arising in *E2a^-/-^* mice have mutations in the PEST domain of NOTCH1 but no mutations have been identified in the HD domain (20). How then is NOTCH1 activated in these leukemias? Insight into this question came when it was revealed that alternative transcription initiation sites are used at the *Notch1* gene in *Ikzf1* ^-/-^ and *E2a* ^-/-^ leukemias (104, 105). Multiple transcripts were identified that initiate from a cryptic promoter upstream of exon 26 leading to a protein that lacks the extracellular domain of NOTCH1. This cyptic promoter can be activated by deletion of the promoter upstream of exon 1, which occurs through a RAG1-dependent mechanism (106). Surprisingly, in *Ikzf1^-/-^* mice, deletion of the first exon of *Notch1* did not impact T cell development, unlike what is seen in *Ikzf1^+/+^* mice, due to use of this alternative mechanism for transcribing *Notch1* in the absence of IKAROS. *Ikzf1^-/-^* thymocytes have increased histone acetylation near IKAROS binding sites located near the alternative *Notch1* promoter raising the possibility that IKAROS represses the use of this alternative mechanism through epigenetic modification (104, 105). Potential E2A binding sites are also present within the alternate promoter and these alternative NOTCH1 isoforms are expressed in *E2a^-/-^* leukemias indicating that E proteins may cooperate with IKAROS to repress alternative promoter use (104). While E2A may repress the alternative promoter, E2A promotes *Notch1* expression in thymic seeding progenitors (42). This deficiency in NOTCH1 could provide a strong selective pressure for NOTCH1 mutation or altered transcription initiation site used to support T cell development. Genomic deletions have also been found in mouse leukemic cells that result in splicing of Exon 1 to downstream exons and again result in proteins that lack the extracellular domain and are constitutively active but dependent on γ-secretase (8, 106).

The mechanisms by which NOTCH1 promotes leukemogenesis have been studied intensively. Interestingly, Notch signaling can impact expression of E2A, at least in mice, where it has been shown that mitogen activated protein kinase phosphorylation of E2A leads to NOTCH1-dependent ubiquitination and proteasomal degradation of E2A (107, 108). Whether this mechanism contributes to ICN induced leukemogenesis in mice or humans requires further investigation. In human T-ALL, a major target of Notch signaling is *c-*MYC, which itself is oncogenic in T lymphocyte progenitors (8, 109). ICN binds to an enhancer 140 Mb downstream of c-*MYC*, whose activity correlates with responsiveness to NOTCH1 inhibitors (110). Moreover, mutation of this enhancer prevents leukemogenesis by ectopic expression of ICN demonstrating that it is an essential target. This enhancer is also regulated by NOTCH3 in NOTCH3-dependent leukemias (111). It is likely that there are many essential targets of NOTCH1 in T-ALL. Indeed, in *E2a^-/-^* leukemias c-MYC expression is stably amplified through trisomy at chromosome 15 and therefore does not require ICN for expression yet these leukemias are still dependent on Notch1 signaling (16, 52).

ChIP-seq analysis for ICN has revealed multiple novel targets of NOTCH1 in leukemia (112). ICN bound regions are in close proximity to RUNX, ETS, and ZNF143 binding motifs and these regions have extensive histone acetylation and H3K4me3 chromatin modifications, indicative of open chromatin and active gene transcription (60, 112). Thus, it is possible that NOTCH1 promotes accessibility to target gene regulatory regions, which allows other T cell specific transcription factors or DNA transcriptional machinery to bind and promote gene expression. Consistent with this idea, NOTCH1 was required for recruitment of RUNX1 and MYB to enhancers located within the *TCRG* and *TCRB* locus (113).

Many ICN target genes, including *DTX1*, *IGF1R*, *IL7R*, and *GIMAP*, have been identified by evaluating changes in gene expression after treatment of leukemias with γ-secretase inhibitors (60, 109). Importantly, many of these genes are co-regulated by T cell specific factors like RUNX and ETS1. Deletion of RUNX1 in DN2/3 thymocytes impairs IL7R expression (114), and expression of dominant-negative RUNX1 and NOTCH1 inhibitors (RUNT and DN-MAML, respectively) suppressed *IL7R* mRNA expression (60). Further, ETS1 binds to multiple NOTCH1 occupied sites in T-ALL (89). Indeed, mice overexpressing NOTCH1 fail to develop leukemia when lacking functional ETS1 suggesting that both of these factors are required for leukemia initiation. ETS1 is frequently over expressed in human T-ALL samples and cell lines indicating that ETS1 may act in concert with NOTCH1 in the human disease as well. Indeed, shRNA–mediated knockdown of ETS1 in human T-ALL lines promoted cell death and significantly down-regulated expression of the oncogenes c-*MYC* and *IGFR1*, as well as other NOTCH1 target genes like *HES1* and *DELTEX1* (89). Understanding of the spectrum of genes induced by NOTCH1 and identifying co-regulators may reveal mechanisms that could be targeted for treatment of T-ALL.

## GATA3

GATA3 is essential for T cell specification and in its absence multipotent progenitors fail to generate committed T lymphocytes (115–117). Ectopic expression of GATA3 can also derail T cell development and force T cell progenitors down alternative lineages, such as the mast cell lineage (118). However, transgenic expression of GATA3, under the control of the CD2 promoter, which drives expression in all lymphocytes, predisposes mice to develop T-ALL-like disease with trisomy of chromosome 15 and activation of NOTCH1, similar to what is seen in *E2a^-/-^* leukemias, although with longer latency (47, 119). GATA3 is elevated in *E2a^-/-^* T cell progenitors and has a negative impact on the ability of DN2 cells to generate T lineage-restricted cells (43). These findings support a role for GATA3 in T cell leukemogenesis and implicate it as a potential contributing factor to T cell transformation in *E2a^-/-^* mice even though this has not been formally demonstrated. Indeed, in non-ETP-ALL GATA3 expression is elevated compared to T cells from healthy donors and defines a stem-like progenitor (120). The mechanism by which GATA3 promotes thymocyte transformation and leukemia survival is not well understood. One potential mechanism involves GATA3’s association with TAL1 as a member of the oncogenic TAL1 complex. Indeed, siRNA knockdown of GATA3 in T-ALL cells represses transcription of TAL1 target genes suggesting that GATA3 is required for proper TAL1 complex function (83). GATA3 and other members of the TAL1 oncogenic complex also bind to the NOTCH1-regulated enhancer downstream of c-*Myc* (90, 110). Mutating the GATA3 binding sites in this enhancer impacted nucleosome eviction and chromatin accessibility, resulting in decreased c-MYC expression and abrogated leukemia development in mice (90). These observations indicate that GATA3 cooperates with TAL1 and NOTCH1 to promote transformation through regulation of c-*MYC*.

In contrast to these cases of increased GATA3 expression, 5% of T-ALL patients have silencing mutations in the *GATA3* gene (1). Consistent with this, another study found that 33% of patients in their cohort with the ETP-ALL subtype had reduced GATA3 expression associated with increased methylation throughout the *GATA3* gene (120). Thus, GATA3 may play multiple distinct roles in T-ALL development, suppressing ETP-ALL or promoting T-ALL at later stages. Decreased GATA3 expression in ETP-ALL is consistent with GATA3’s function in promoting T cell lineage differentiation as *GATA3* silencing could contribute to a developmental block at the ETP stage that supports transformation. It also seems likely that GATA3 is not a driver mutation and its function may be dependent on the spectrum of additional mutations that occur during transformation.

## LEF1/TCF1

The NOTCH1 target gene *Tcf7*, encoding the protein TCF1, is also implicated as a suppressor of T cell transformation (57, 121). TCF1 is a member of the HMG box family of proteins along with the closely related protein LEF1. Both TCF1 and LEF1 can promote transcription in response to canonical WNT signaling activation or repress transcription through recruitment of the Groucho related co-repressors such as TLE3 (122, 123). In the absence of TCF1, thymocytes have a developmental block at the ETP, DN2, and ISP stages whereas mice lacking LEF1 have no obvious defects in DN thymocytes (54–56, 124). Combined deletion of *Tcf7* and *Lef1* exacerbates the phenotype seen in *Tcf7*-deficient mice, leading to a nearly complete block in T cell development (54). This observation indicates that TCF1 and LEF1 have overlapping functions and that LEF1 partially compensates for the loss of TCF1. In addition to the defects seen in T lymphopoiesis, approximately 50% of *Tcf7^-/-^* mice develop T-ALL (57, 121). *Tcf7^-/-^* leukemias are heterogeneous; phenotypically resembling DN3, DN4, and DP thymocytes. Despite this cell surface phenotype, RNA profiling revealed that the transcriptome of *Tcf7^-/-^* T-ALLs is related to that of human ETP-ALLs, which is consistent with the early requirement for TCF1 in T cell development (57). *Tcf7^-/-^* leukemias have activated NOTCH signaling and inhibiting this pathway with GSI at least partially impacts their viability (121). Further, *Tcf7^-/-^* leukemias highly express ID2 and LEF1, particularly in a subset of T cell progenitors with a gene signature predictive of high leukemic potential, suggesting that that suppression of E protein activity may be a feature of transformation in this model (57, 58). Indeed, *Tcf7^-/-^Id2^-/-^* mice showed an increased latency of leukemogenesis consistent with this hypothesis.

Like *Tcf7^-/-^* leukemias, *E2a^-/-^* leukemias have high expression of *Lef1* and LEF1 is required for the survival and proliferation of these leukemias (52). LEF1 is an oncogene in acute myeloid leukemia and in multiple forms of B lymphocyte leukemia and it is suppressed by TCF1 (125–128). Ectopic expression of LEF1 in HSCs induced acute myeloid leukemia-like or B cell ALL-like disease in mice, demonstrating LEF1’s oncogenic potential (127). In an adult cohort of T-ALL patients, high LEF1 expression was associated with increased expression of the oncogenes encoding c-MYC and CYCLIN D1 suggesting that LEF1 is positively associated with T cell leukemia (129). Moreover, 4 unique mutations that augment LEF1 function were found in these patients. In contrast, approximately 11% of pediatric T-ALL patients were found to have inactivating mutations in the *LEF1* gene (7, 130). These mutations consist of deletions or truncation mutations, both resulting in lower LEF1 function. These conflicting findings suggest that LEF1 can play multiple roles in T cell leukemia. Indeed, while *E2a^-/-^* leukemias are dependent on LEF1, inactivation of *Lef1* in *E2a^-/-^* mice prior to transformation did not prevent transformation; rather, it reduced leukemia latency and resulted in leukemias with a unique gene expression program compared to *E2a^-/-^* leukemias (53). Taken together, these experiments reveal that the timing of genetic alterations in the evolution of T-ALL can determine latency, phenotype and genetic susceptibilities within these cells.

## Conclusions

T-ALL is a heterogeneous disease that is associated with mutations in numerous T cell specific transcription factors, epigenetic regulators, and signaling pathways. Despite this heterogeneity, many of the mutations seen in patients impact the function of E proteins and their target genes. Recent studies looking at the clonal evolution of T-ALL have further indicated that TAL1 upregulation is a founding event in the human disease, preceding mutations in NOTCH1 (131). Thus, understanding how reduced E protein function impacts T cell development and leukemogenesis is highly relevant to the human disease. The *E2a^-/-^* mouse model has revealed connections between T cell developmental alterations and the transformation process. For example, *Notch1*, *Gata3*, and *Lef1/Tcf7* expression are altered early in T cell development in *E2a^-/-^* mice and contribute to transformation. The pathways regulated by these proteins are promising candidates for therapeutic intervention in T-ALL. Indeed, inhibitors of NOTCH1 activation or function have been in clinical trials, however many of these inhibitors have adverse side effects due to the many cell types that rely on Notch signaling (11, 132). Novel NOTCH1 inhibitors that prevent the formation of the ICN transcriptional activation complex show decreased off target effects *in vivo* compared to inhibitors that target NOTCH1 activation, but still induce mild intestinal toxicity (133). Further investigation of oncogenic and tumor suppressive pathways in murine T-ALL models, including GATA3 and TCF1/LEF1, and their application to human leukemia may identify novel targets that alone, or in combination with other targets, will have fewer side effects without sacrificing anti-leukemia efficacy.

## Author Contributions

GP and BK wrote the manuscript and approved the final version. All authors contributed to the article and approved the submitted version.

## Funding

Our studies on *E2a^-/-^* T cell leukemia were supported by the NIH/NIAID (R01 AI106352, R21 AI119894, R21 AI096350, and R03 AI137605) and the Janet Rowley Fund (University of Chicago Comprehensive Cancer Center).

## Conflict of Interest

The authors declare that the research was conducted in the absence of any commercial or financial relationships that could be construed as a potential conflict of interest.

## Publisher’s Note

All claims expressed in this article are solely those of the authors and do not necessarily represent those of their affiliated organizations, or those of the publisher, the editors and the reviewers. Any product that may be evaluated in this article, or claim that may be made by its manufacturer, is not guaranteed or endorsed by the publisher.
